# Risk of stroke in chronic heart failure patients with preserved ejection fraction, but without atrial fibrillation: analysis of the CHARM-Preserved and I-Preserve trials

**DOI:** 10.1093/eurheartj/ehw509

**Published:** 2016-11-13

**Authors:** Azmil H. Abdul-Rahim, Ana-Cristina Perez, Rachael L. MacIsaac, Pardeep S. Jhund, Brian L. Claggett, Peter E. Carson, Michel Komajda, Robert S. McKelvie, Michael R. Zile, Karl Swedberg, Salim Yusuf, Marc A. Pfeffer, Scott D. Solomon, Gregory Y.H. Lip, Kennedy R. Lees, John J.V. McMurray, on behalf of the Candesartan in Heart failure Assessment of Reduction in Mortality and Morbidity-Preserved (CHARM-Preserved) and the Irbesartan in Heart Failure with Preserved Systolic Function (I-Preserve) Steering Committees

**Affiliations:** 1 BHF Glasgow Cardiovascular Research Centre, Institute of Cardiovascular and Medical Sciences, University of Glasgow, Glasgow G12 8TA, UK; 2 Cardiovascular Division, Brigham and Women’s Hospital, Boston, MA, USA; 3 Division of Cardiology, The VeteransAffairs Medical Center, Washington, DC, USA; 4 Department of Cardiology, University Pierre and Marie Curie, Paris, France; 5 Population Health Research Institute, McMaster University, Hamilton, ON, Canada; 6 Division of Cardiology, Medical University of South Carolina, Charleston, SC, USA; 7 Department of Molecular and Clinical Medicine, University of Gothenburg, Göteborg, Sweden; 8 National Heart and Lung Institute, Imperial College, London, UK; 9 Hamilton Health Sciences, Hamilton, ON, Canada; 10 University of Birmingham Institute of Cardiovascular Sciences, City Hospital, Birmingham, UK

**Keywords:** Heart failure with preserved ejection fraction, Stroke, Risk-factors

## Abstract

**Aims:**

The incidence and predictors of stroke in patients with heart failure and preserved ejection fraction (HF-PEF), but *without* atrial fibrillation (AF), are unknown. We described the incidence of stroke in HF-PEF patients with and without AF and predictors of stroke in those without AF.

**Methods and results:**

We pooled data from the CHARM-Preserved and I-Preserve trials. Using Cox regression, we derived a model for stroke in patients without AF in this cohort and compared its performance with a published model in heart failure patients with reduced ejection fraction (HF-REF)—predictive variables: age, body mass index, New York Heart Association class, history of stroke, and insulin-treated diabetes. The two stroke models were compared and Kaplan–Meier curves for stroke estimated. The risk model was validated in a third HF-PEF trial. Of the 6701 patients, 4676 did not have AF. Stroke occurred in 124 (6.1%) with AF and in 171 (3.7%) without AF (rates 1.80 and 1.00 per 100 patient-years, respectively). There was no difference in performance of the stroke model derived in the HF-PEF cohort and the published HF-REF model (*c*-index 0.71, 95% confidence interval 0.57–0.84 vs. 0.73, 0.59–0.85, respectively) as the predictive variables overlapped. The model performed well in the validation cohort (0.86, 0.62–0.99). The rate of stroke in patients in the upper third of risk approximated to that in patients with AF (1.60 and 1.80 per 100 patient-years, respectively).

**Conclusions:**

A small number of clinical variables identify a subset of patients with HF-PEF, but without AF, at elevated risk of stroke.

## Introduction

Up to half of patients with heart failure have a preserved ejection fraction (HF-PEF).^1–3^ These patients differ from heart failure patients with reduced ejection fraction (HF-REF) in several aspects—they tend to be older, are more often women, and are more likely to have a history of hypertension and atrial fibrillation (AF); they are less likely to have coronary artery disease. Although, mortality rates may not be as high as in patients with HF-REF, the prognosis of HF-PEF patients is considerably worse than that of patients with hypertension, angina pectoris, AF, or diabetes in the same age range and gender distribution.^4^ The single most common cause of hospital admission in these patients is worsening heart failure and this, along with death, has been the focus of therapeutic interventions in HF-PEF.^4^ However, given the demographic profile and co-morbidity cluster characterizing these patients, stroke may also be a clinically important outcome in HF-PEF. Little is known about the incidence of stroke in HF-PEF, particularly in the absence of AF. 

To investigate this further, we therefore combined and analysed patient-level data from two large HF-PEF trials, the Candesartan in Heart failure Assessment of Reduction in Mortality and Morbidity-Preserved trial (CHARM-Preserved, ClinicalTrials.gov NCT0 0634712)^5^ and the Irbesartan in Heart Failure with Preserved Systolic Function trial (I-Preserve, NCT00095238),^6^ to provide a robust estimate of the current incidence of stroke in patients with HF-PEF, with and without AF. We also tested a simple clinical model, developed in HF-REF,^7^ for predicting the risk of stroke in patients *without* AF in this pooled dataset. Easy identification of those at highest risk of stroke coupled with the availability of new oral anticoagulants with a low risk of bleeding might allow for a stroke prevention strategy which has an acceptable benefit/risk balance in patients with HF without AF.

## Methods

### Trial patients

In order to have a sufficiently large number of HF-PEF patients without AF for analysis, we pooled data from the CHARM-Preserved (NCT00634712) and I-Preserve (NCT00095238) trials. Each was a randomized, double-blind, placebo-controlled, multicentre trial and was approved by the appropriate institutional review boards. CHARM-Preserved and I-Preserve enrolled 3023 and 4128 patients, respectively.^5^^,^^6^ Together, these trials included a broad spectrum of patients with chronic HF-PEF.

CHARM-Preserved enrolled patients aged ≥18 years in New York Heart Association (NYHA) functional class II–IV with a left ventricular ejection fraction (LVEF) >40% (although for the purposes of this study we included only patients with an LVEF ≥45%). I-Preserve enrolled patients aged ≥60 years in NYHA functional class II–IV with an LVEF ≥45% and corroborating ECG, echocardiographic or radiologic evidence. In addition, patients must have been hospitalized for heart failure in the preceding 6 months or, if not, had to be in NYHA functional class III or IV. N-terminal pro B-type natriuretic peptide (NT-proBNP) was measured at baseline in I-Preserve but not in CHARM-Preserved. In CHARM-Preserved, patients were randomly assigned to candesartan (target dose of 32 mg once daily) or matching placebo.^5^ In I-Preserve, patients were randomized to irbesartan (target dose 300 mg once daily) or matching placebo.^6^ The primary outcome in CHARM-Preserved was the composite of cardiovascular death or HF hospitalization^5^^,^^8^ and in I-Preserve it was the composite of all-cause mortality or cardiovascular hospitalization.^6^^,^^9^ The median follow-up in CHARM-Preserved was 3.1 years and in I-Preserve it was 4.1 years. Study treatment did not reduce the risk of the primary outcome or the risk of stroke in the either trial.^5^^,^^6^

### Incident stroke

Incident strokes were centrally adjudicated by an independent endpoint committee in each trial using similar definitions and stroke was part of the primary or secondary composite cardiovascular outcomes in both trials.^5^^,^^6^^,^^8^^,^^9^ Stroke in both trials was defined as a persistent (≥24 h) disturbance of focal neurological function resulting in symptoms thought to be due to cerebral infarction, evidence of haemorrhage or for which there is no certain aetiology.^5^^,^^6^^,^^8^^,^^9^

### Incident atrial fibrillation

The occurrence of AF was retrospectively collected in CHARM-Preserved during the trial close-out using a specifically designed case-report form. Incident AF was recorded prospectively in I-Preserve, using a specific case-report form.

### Statistical methods

We included only patients with an LVEF of ≥45% (all 4128 patients in I-Preserve and 2573 of the 3023 in CHARM-Preserved). Patients with AF were defined as those with either AF confirmed on their baseline ECG or a history of AF. The remaining patients were defined as those ‘without AF’. Descriptive statistics were used to describe the pooled patient population from both trials and to compare these two subgroups, using means (standard deviation) or medians [inter-quartile range (IQR)] for continuous variables and count (percentage) for categorical variables.

The incidence rate of stroke (per 100 patient-years) was calculated over the trial follow-up period and was compared among the AF and no AF subgroups. We plotted Kaplan–Meier (KM) curves for the occurrence of stroke, according to AF status. To satisfy the assumption of the independence of stroke events, recurrent stroke events in a patient after randomization were not included in the analysis.

Continuous variables [e.g. body mass index (BMI), ejection fraction, and creatinine level] were assessed by visual inspection of restricted cubic splines to identify potential non-linear effects. Uni- and multivariable predictors of the risk for stroke were evaluated using Cox proportional hazards regression analysis in patients without AF. Two separate multivariable analyses for stroke were created. First, an ‘HF-PEF stroke model’ was created using established predictors of ischaemic stroke^10^^–^^15^ with the addition of variables that were significant (*P* < 0.05) in univariable analysis of our dataset. The final list of variables included was age, sex, LVEF, NYHA class III/IV, BMI, creatinine level, systolic blood pressure, history of stroke, hypertension, and diabetes treated with insulin. Second, we applied a recently published multivariable predictive model for stroke in patients with HF-REF (HF-REF stroke model) in our HF-PEF cohort.^7^ The five variables included in this model were age, BMI, NYHA class, history of stroke, and diabetes treated with insulin. There were no data missing for the baseline variables used either model. We calculated the hazard ratio and corresponding 95% confidence intervals (95% CI) to express the hazard rate of stroke. The statistical contribution of each variable to the predicted risk of stroke was assessed by the χ^2^ statistic. In order to be consistent with our previous publication,^7^ we compared each model’s discrimination ability using estimates of overall *c*-index for the Cox regression models according to the method of Pencina and D’Agostino,^16^ as outlined by Liu *et al.*^17^ We pre-determined that we would proceed using only the HF-REF stroke model if the overall *c*-indexes for the two models were not meaningfully different.

The coefficients from statistically significant variables in the final multivariable model were used to calculate an individual patient’s risk score for stroke. The KM curves for occurrence of stroke according to tertiles of risk score were plotted.

Final model calibration and the ability to separate patients into risk groups were assessed by observing predicted compared with observed outcomes in terciles, and by using the Hosmer–Lemeshow goodness-of-fit test. The model’s discrimination abilities were evaluated by the overall *c*-index.^16^^,^^17^

We also conducted a sensitivity analysis to compare the cumulative incidence function for stroke estimated using competing risk technique (to account for the competing risk of death)^19^^,^^20^ with the rates of stroke described from the traditional KM curves above. We also compared the overall *c*-index of the model with the traditional Harrell’s *c*-statistic.^16–18^

Finally, we validated the preferred risk model in a third HF-PEF trial: the Treatment of Preserved Cardiac Function Heart Failure with an Aldosterone Antagonist (TOPCAT) (NCT00094302).^21^ TOPCAT included patients aged ≥50 years with at least one symptom and sign of heart failure, an LVEF ≥45% and either a hospitalization with heart failure in the preceding 12 months or an elevated NT-proBNP or BNP.

All analyses were undertaken using SAS version 9.3 (SAS Institute, Inc., Cary, NC, USA).

## Results

Of the 6701 patients with an LVEF ≥45%, 2025 (30%) had a history of AF or AF on their baseline ECG and 4676 patients (70%) had no AF.

### Baseline characteristics

The baseline characteristics of patients with and without AF are shown in the Supplementary material online, *TableS1*. The baseline characteristics of patients without AF, according whether or not they experienced a subsequent stroke, are shown in *Table **1*.

**Table 1:** Baseline characteristics according to stroke outcome in patients without AF

	Patients without AF (*N*=4676)	**Non-stroke** (*n*=4505)	**Stroke** (*n*=171)
Demographics, *n* (%)			
Age, year	69 ± 9	69 ± 9	71 ± 8
<65	1400 (30)	1366 (30)	34 (20)
65 to < 75	2032 (43)	1956 (43)	76 (44)
≥75	1244 (27)	1183 (26)	61 (36)
Race			
Caucasians	4273 (91)	4116 (91)	157 (92)
Afro-American/Afro-Caribbean	155 (3)	148 (30)	4 (7)
Other	248 (5)	241 (5)	7 (4)
Female sex	2542 (54)	2459 (55)	83 (49)
NYHA class			
II	1657 (35)	1612 (36)	42 (26)
III	2918 (62)	2799 (62)	119 (70)
IV	101 (2)	94 (2)	7 (4)
Duration of heart failure, year			
<2	2778 (59)	2673 (59)	105 (61)
2–5	1110 (24)	1076 (24)	34 (20)
>5	764 (16)	734 (16)	30 (18)
LV ejection fraction, %	58 ± 9	58 ± 9	57 ± 8
Baseline vital signs			
BMI, kg/m^2^	30 ± 6	30 ± 6	29 ± 5
BP, mmHg			
Systolic	137 ± 16	137 ± 16	140 ± 15
Diastolic	79 ± 10	79 ± 10	79 ± 9
Pulse pressure	58 ± 14	58 ± 14	61 ± 14
Heart rate, b.p.m.	71 ± 11	71 ± 11	71 ± 10
Laboratory measurements			
Serum creatinine, µmol/L	88 ± 29	88 ± 29	96 ± 33
Haemoglobin, g/dL	14 ± 2	14 ± 2	14 ± 1
NT-proBNP^a^, pg/mL(median ± IQR)	230 (104–537)	225 (104–525)	426 (170–1121)
Medical history, *n* (%)			
Coronary heart disease	2960 (63)	2855 (63)	105 (61)
Myocardial infarction	1599 (34)	1534 (34)	65 (38)
Angina pectoris	2517 (54)	2429 (54)	88 (51)
CABG or PCI	1078 (23)	1044 (23)	34 (20)
Hypertension	3779 (81)	3632 (81)	147 (86)
Diabetes mellitus	1313 (28)	1245 (28)	68 (40)
Stroke	379 (8)	343 (8)	36 (21)
ICD	11 (0.2)	11 (0.2)	0 (0)
Current smoker	2597 (56)	2502 (56)	95 (56)
Medication, *n* (%)			
Diuretic (loop or thiazide)	3392 (73)	3266 (73)	126 (74)
Loop diuretic	2278 (49)	2195 (49)	83 (49)
Thiazide diuretic	1481 (32)	1430 (32)	51 (30)
ACE inhibitor	1020 (22)	982 (22)	38 (22)
Aldosterone antagonist	788 (17)	753 (17)	35 (20)
Beta-blocker	2761 (59)	2663 (59)	98 (57)
Digitalis glycoside	405 (9)	391 (9)	14 (8)
Calcium channel blocker	1809 (39)	1747 (39)	62 (36)
Antiarrhythmic drug	179 (4)	173 (4)	6 (4)
Long-acting nitrate	1476 (32)	1410 (31)	66 (39)
Lipid lowering therapy	1786 (38)	1734 (38)	52 (30)
Antiplatelet therapy	3204 (69)	3080 (68)	124 (73)
Anticoagulant therapy	263 (6)	255 (6)	8 (5)
Any antihthrombotic (antiplatelet or anticoagulant therapy)	3408 (73)	3278 (73)	130 (76)
Antidiabetic therapy	1096 (23)	1040 (23)	56 (33)
Insulin therapy	438 (9)	409 (9)	29 (17)
Placebo arm in the original trial	2322 (50)	2231 (50)	91 (53)

All continuous values are given in mean ±  standard deviation unless stated otherwise.

AF, atrial fibrillation; *n*  (%), number of observations  (percentage of observations within the group); BMI, body mass index; BP, blood pressure; NYHA, New York Heart Association; NT-proBNP, N-terminal pro B-type natriuretic peptide; CABG, coronary artery bypass graft; PCI, percutaneous coronary intervention; ICD, implantable cardioverter defibrillator; ACE, angiotensin converting enzyme.

a Available in 2452 patients.

**Table 2:** List of variables from the ‘HF-REF model for stroke’

Variables	Coefficients from HF-REF stroke model^7^
Previous Stroke	0.591
Diabetes treated with insulin	0.626
Age (per 10 years increase)	0.331
BMI (per 5 kg/m^2^ increase up to 30)	−0.301
NYHA class (NYHA III and IV)	0.472

See the Supplementary material for explanation of how to use coefficients of the variables to calculate individual patient’s risk score of stroke.

HF-REF, heart failure with reduced ejection fraction; BMI, body mass index; NYHA, New York Heart Association.

#### Patients with and without atrial fibrillation

Patients *without* AF were younger and were more likely to have a history of coronary artery disease and hypertension, compared with patients *with* AF. Patients without AF also had a slightly higher systolic blood pressure but had a lower mean serum creatinine and much lower median NT-proBNP level than patients with AF. There were also notable differences in medical therapy, particularly in use of antiplatelet therapy (69% of patients without AF vs. 39% of those with AF) and anticoagulant treatment (6% vs. 57%, respectively), but also in relation to diuretics, mineralocorticoid receptor antagonists, antiarrhythmic agents, and digoxin.

#### Patients without atrial fibrillation**—with and without incident stroke during follow-up**

Among patients without AF, those who experienced a stroke (compared with those who did not) were older, more likely to have a history of diabetes, hypertension, and stroke and had worse NYHA functional class. Patients experiencing stroke also had a higher systolic blood pressure, creatinine, and NT-proBNP level. Compared with those not experiencing stroke, those who did were less likely to be treated with lipid lowering therapy but more likely to be taking nitrates, anti-platelet therapy, and insulin. Very few patients in either group were treated with an oral anticoagulant (263 in total, 6%). LVEF did not differ between patients with and without stroke.

### Rates of stroke

#### **Patients with a**trial fibrillation

**Figure 1:**
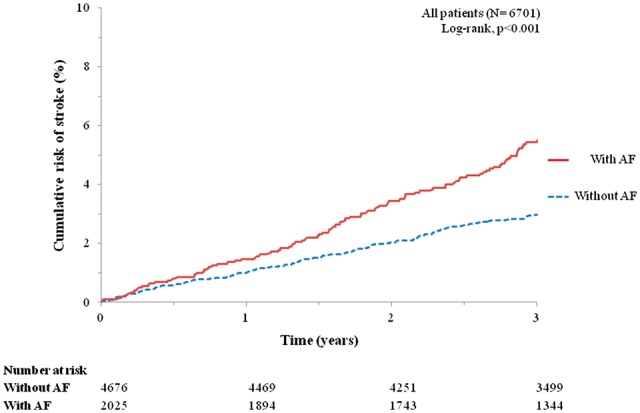
Kaplan–Meier plot stroke for chronic heart failure patients with preserved ejection fraction according to atrial fibrillation status at baseline. AF, atrial fibrillation.

The median follow-up time in patients with AF was 3.4 (IQR: 2.8–4.4) years and 124 of these 2025 patients (6.1%) experienced a stroke (1.80 per 100 patient-years). The 1, 2, and 3 year KM rates for stroke were 1.5 (95% CI: 1.0–2.1)%, 3.5 (95% CI: 2.7–4.4)%, and 5.5 (95% CI: 4.5–6.6)%, respectively (*Figure **1*). The stroke rate in patients treated with an anticoagulant was 1.51 per 100 patient-years; and in those not treated with an anticoagulant it was 2.19 per 100 patient-years (yearly rates shown in the Supplementary material online, *FigureS1*).

#### **Patients without a**trial fibrillation

The median follow-up time in patients without AF was 3.5 (IQR: 3.0–4.6) years and 171 of these 4676 patients (3.7%) experienced a stroke (1.00 per 100 patient-years). The 1, 2, and 3 year KM rates of stroke were 1.0 (95% CI: 0.8–1.4)%, 2.0 (95% CI: 1.7–2.5)%, and 3.0 (95% CI: 2.5–3.5)%, respectively (*Figure **1*).

#### Incident AF and risk of stroke

In CHARM-Preserved, 1781 patients did not have AF at baseline. Out of 1781, 59 patients (3.3%) experienced a stroke. Of these 59 patients, 10 (17%) developed new AF before the occurrence of their stroke; the number of patients with a stroke without preceding AF was 49 (83%). Development of AF reported was not reported in any patient following a stroke.

In I-Preserve, 2895 patients did not have AF at baseline. Out of 2895, 112 patients (4%) experienced a stroke. Of these 112 patients, 18 (16%) developed new AF before the occurrence of their stroke; the number of patients with a stroke without preceding AF was 94 (84%). Twenty patients (18%) with an incident stroke had new AF reported before or after their stroke.

### Predictors of stroke in patients with heart failure and preserved ejection fraction *without* AF

**Figure 2:**
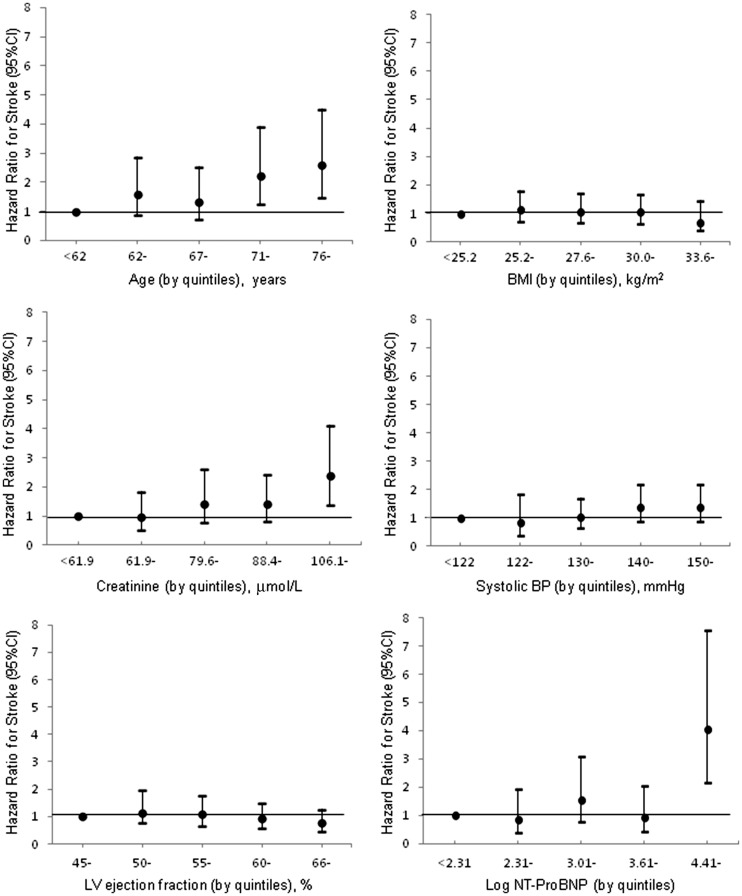
The relationship between baseline variables and risk of stroke in patients with heart failure and preserved ejection fraction *without* atrial fibrillation. Variables are divided by quintiles. BMI, body mass index; BP, blood pressure; LV, left ventricular; NT-proBNP, N-terminal pro-B-type natrieretic peptide.

*Figure* *2* and Supplementary material online, *TableS2* show the relationship between baseline variables and risk of stroke (univariable analysis). Supplementary material online, *TableS3* shows an adjusted analysis using the four independent predictors identified in a multivariable stroke model developed in the present *HF-PEF* cohort (previous stroke, age, diabetes treated with insulin, and male sex). These overlapped with the five independent predictors in the *HF-REF* stroke model (previous stroke, age, diabetes treated with insulin, BMI, and NYHA class) (Table 2). The overall *c*-index for the HF-PEF model was 0.71 (95% CI: 0.57–0.84) compared with 0.73 (0.59–0.85) using the HF-REF model (*P*-value for difference = 0.415). Thus, we proceeded using the previously validated HF-REF model. This model can be used to calculate an individual’s risk of stroke as described in the Supplementary material online, *Supplementary material*.

**Figure 3:**
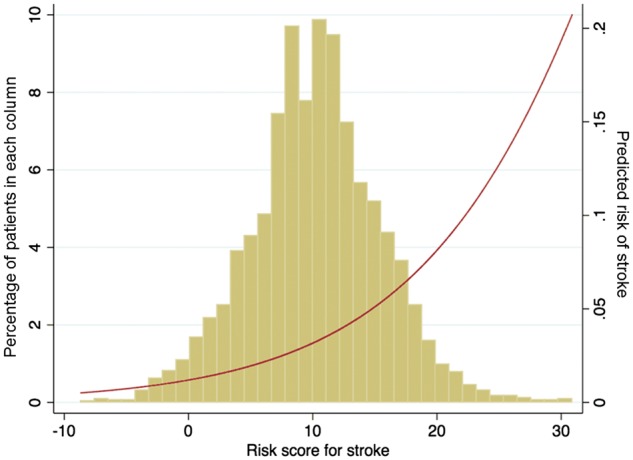
Distribution of risk score for stroke and its relation to predicted risk of stroke within the follow-up period.

**Figure 4:**
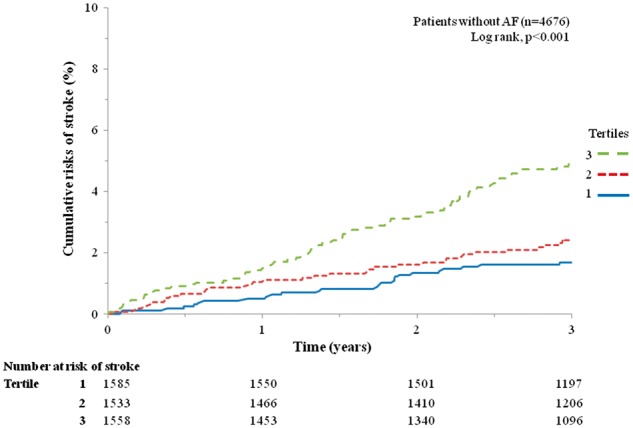
Kaplan–Meier plot for stroke according to tertile of risk score in patients without atrial fibrillation.

*Figure* *3* shows the distribution of the risk score for stroke and illustrates the risk of stroke for a given score. A score of approximately 12 predicts a risk of stroke similar to that which was seen among patients with AF in the current cohort. *Figure **4* shows KM curves for stroke with patients classified into three equal-sized groups according to risk score. The number of strokes in tertiles 1, 2 and 3 were 37, 45 and 89, respectively. The 1, 2 and 3 year KM rates of stroke in the two higher risk tertiles were tertile 2: 1.1 (95% CI: 0.7–1.7)%, 1.6 (95% CI: 1.1–2.4)%, and 2.4 (95% CI: 1.7–3.3)%, respectively; and tertile 3: 1.4 (95% CI: 1.0–2.2)%, 3.2 (95% CI: 2.4–4.2)%, and 4.9 (95% CI: 3.9–6.2)%, respectively (*Figure **4*). Patients in risk-tertile 3 had an overall stroke rate of 1.60 per 100 patient-years.

**Figure 5:**
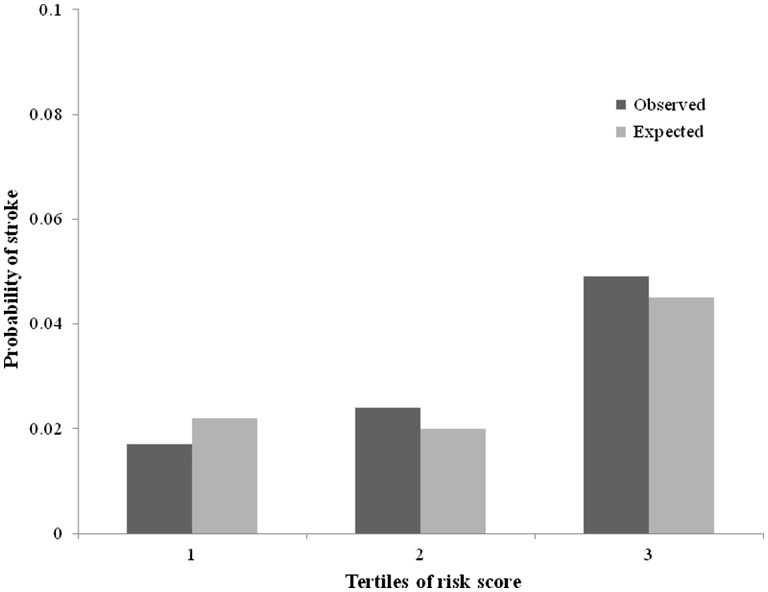
Comparison of observed and expected stroke rates after 3 years for patients categorized by tertile of risk-score derived from the heart failure with reduced ejection fraction stroke model.^7^ Observed shows the 3 year Kaplan–Meier rate for each tertile; expected shows estimate from the Cox model for each tertile.

*Figure* *5* shows the model’s goodness-of-fit by comparing observed and expected probabilities of stroke at 3 years with the patients divided into tertiles. The calibration was also assessed using the Hosmer–Lemeshow test, which was *P* = 0.761.

### Validation of stroke risk model

We tested the predictive model in TOPCAT, which included 1240 patients with and 2205 patients without AF. The mean follow-up was 3.5 years. There were 65 strokes in the patients with AF and 52 strokes in those without AF, giving stroke rates in patients with and without AF 1.64 and 0.71 per 100 patients-years, respectively.

Using the same analytical approach (see Supplementary material online, *TableS4*, *FiguresS2andS3*), the 1, 2 and 3 year KM rates of stroke in patients without AF, in the two higher risk tertiles were: tertile 2: 1.3 (95% CI: 0.6–2.4)%, 1.3 (95% CI: 0.6–2.3)% and 1.7 (95% CI: 0.9–2.9)%, respectively; and tertile 3: 1.5 (95% CI: 0.8–2.7)%, 1.7 (95% CI: 0.9–2.9)% and 2.6 (95% CI: 1.6–4.1)%, respectively. Patients in risk-tertile 3 of the validation model derived from TOPCAT cohort had an overall stroke rate of 1.06 per 100 patient-years. The overall *c*-index for the model was 0.86 (95% CI: 0.62–0.99).

Sensitivity analysis that evaluated the cumulative incidence functions of stroke for the corresponding KM curves reported above is available in the Supplementary material online, *FiguresS4–S7*. There is little difference between the two types of curves. The comparison for the ‘stroke in HF-REF’ model’s discrimination ability within the HF-PEF cohort using the overall *c*-index and the traditional Harrell’s *c*-method is available in the Supplementary material online, *TableS5*.

## Discussion

In this analysis, HF-PEF patients *with* AF were at a high risk of stroke, with an average incidence rate of 1.8% per year which is similar to that recently reported in HF-REF patients with AF (1.6% per year).^7^

HF-PEF patients *without* AF in this study had a lower risk of stroke compared with those *with* AF. However, the overall rate of stroke in HF-PEF patients *without* AF (1.0% per year) was similar to the rate we recently reported in HF-REF patients without AF (1.2% per year). Moreover, as in HF-REF, a small number of demographic and clinical variables identified a subset of HF-PEF patients without AF who were at greater risk of stroke than the remainder. Specifically, in our pooled analysis, patients in the upper third of the risk score had a rate of stroke (1.6% per year) which was higher than in HF-PEF patients with AF receiving an anticoagulant (1.5% per year), although not as high as in similar patients *not* treated with an anticoagulant (2.2% per year).

We have been unable to find other reports of the risk of stroke in HF-PEF patients *without* AF although in patients in the same age range in clinical trials for hypertension (i.e. with a similar co-morbid phenotype to HF-PEF) have a stroke risk of around 1% per year or less.^22–26^ In HF-PEF patients *with* AF randomized to warfarin in ARISTOTLE^27^ the rate of stroke was 1.4% per year which was similar to the rate in anticoagulant-treated AF patients in our study (1.5% per year). In AF patients with HF and an LVEF >40% in RELY-AF^28^ the rate of stroke or systemic embolism was 2.07% per year in the warfarin group; in ROCKET-AF^29^ the rate of the same outcome in similarly defined patients was 2.06% per year. The higher event rates in the latter two trials are due to broader composite outcome (which included non-cerebral systemic embolism) and the requirement for patients in these trials to have additional risk factors for stroke.

The similar risk of stroke in patients with HF-PEF and HF-REF, *without* AF, is also of interest. We previously reported that LVEF was not predictive of stroke in HF-REF patients without AF. Neither was LVEF an independent predictor of stroke risk in this study although we examined only patients with an LVEF ≥45%. This finding is consistent with observations in three recent trials comparing non-Vitamin K antagonist oral anticoagulants with warfarin in patients *with* AF. In those trials, the risk of stroke and systemic embolism was similar, irrespective of LVEF category, in patients with AF and concomitant HF. A similar conclusion was reached by the Atrial Fibrillation Clopidogrel Trial with Irbesartan for Prevention of Vascular Events (ACTIVE) in AF patients *not* treated with an oral anticoagulant where the risk of stroke was similar in patients with concomitant HF-REF or HF-PEF.^30^

As in HF-REF, we found that neither systolic blood pressure nor history of hypertension was independent predictor of stroke. Although this contrasts with the findings in other patient cohorts, it is consistent with the ‘reverse epidemiology’ of heart failure and the known association between higher blood pressure and better outcomes in this condition.^31–33^ Likewise, we saw an association between lower BMI and higher risk of stroke, another feature of the ‘reverse epidemiology’ in heart failure.^31–33^

A particular strength of this study is the validation of our predictive model in another dataset (TOPCAT). Consequently, our findings have clear clinical implications. With a small number of routinely collected clinical variables it is possible to identify patients with HF-PEF, but without AF, who may be at sufficiently high risk of stroke *potentially* to justify anticoagulation. Clearly, there is as yet no trial evidence to justify such treatment but our findings suggest a means of identifying patients for such a trial. Consistent with this hypothesis, prior trials in patients with heart failure and *reduced* ejection fraction collectively suggest that anticoagulation can reduce the risk of stroke in patients in sinus rhythm. However, in the largest of these, the Warfarin vs. Aspirin in Reduced Cardiac Ejection Fraction trial (WARCEF), although warfarin was effective in reducing ischaemic stroke this benefit was offset by major bleeding. With non-vitamin K oral anticoagulants, the risk-to-benefit balance might be more favourable, especially as the target International Normalized Ratio (INR) in WARCEF was 2.75 (range 2.0–3.5).^34–37^

### Limitations

Each of the two trials included had specific inclusion and exclusion criteria and, hence, our findings may not be generalizable to all patients with HF-PEF. Notably, few patients were in NYHA class IV and worse functional class was a predictor of higher risk of stroke. Hence, the risk of stroke may be higher in ‘real world’ patients than in the cohort studied. Although our data suggest that only the minority of strokes is related to incident AF, systematic detection of new onset AF was insensitive, e.g. continuous ambulatory monitoring was not performed. It is widely recognized that silent AF is frequent in heart failure and undetected AF may have accounted for more strokes than realized. However, waiting for the development of clinically recognized AF before employing anticoagulant therapy may not be the ideal preventive strategy and the best and most cost-effective way to screen for silent AF in HF-PEF is unknown. In addition, these patients may have other reasons to develop thromboembolic and other types of ischaemic stroke, e.g. endothelial dysfunction and blood stasis. Therefore, we believe that our findings support a potential preventive role for anticoagulant therapy in HF-PEF patients in sinus rhythm, particularly as new agents with a lower risk of bleeding are available. Of course, this hypothesis needs to be tested prospectively in a randomized trial and it may be too simplistic to assume that an anticoagulant can substantially reduce the risk of stroke in those with HF-PEF at highest risk.

CHARM-Preserved and I-Preserve were randomized controlled heart failure trials, rather than stroke trials, and used definitions of stroke consistent with other heart failure trials conducted during the same period. Although the definition may not be identical to that used in contemporary stroke trials, it was applied consistently by adjudicators blind to treatment allocation and thus gave an unbiased estimate of treatment effect. Unfortunately, classification of stroke subtype was not carried out in both trials. When the trials were conducted, neuroimaging was not standard in patients with suspected stroke in many, if not most countries, involved. Therefore, we are unable to distinguish between ischaemic and haemorrhagic strokes.

In conclusion, we found that a relatively high-risk subset of a third of HF-PEF patients *without* AF have a risk of stroke similar to that in HF-PEF patients *with* AF. This higher-risk subset can be identified using five simple clinical variables. The risk of stroke is similar in HF-PEF and HF-REF patients without AF and is predicted by the same variables. The risk of stroke in these patients might be reduced by treatment with an oral anticoagulant but this hypothesis needs to be tested in a clinical trial. The rate of stroke in the highest risk tertile was not quite as high as in patients with AF not treated with an anticoagulant so it is uncertain what the benefit/risk ratio of such treatment might be.

## Supplementary material

Supplementary material is available at *European Heart Journal* online.

## Authors’ contributions

A.H.A.R., A.C.P., R.L.M., and B.L.C. performed the statistical analysis; J.J.V.M. and K.R.L. handled funding and supervision; J.J.V.M. and A.H.A.R. conceived and designed the research, and drafted the manuscript; all co-authors made critical revision of the manuscript for key intellectual content.

## Funding

A.H.A.R. received departmental funding to perform the analysis. A-CP was supported by the Medical Research Council award MC_UU_12017/10.

**Conflict of interest:** K.R.L. chairs the Data and Safety Monitoring Board for the RESPECT-ESUS Trial, sponsored by Boehringer Ingelheim. G.Y.H.L. is a consultant for Bayer/Janssen, Astellas, Merck, Sanofi, BMS/Pfizer, Biotronik, Medtronic, Portola, Boehringer Ingelheim, Microlife and Daiichi-Sankyo, and he is a speaker for Bayer, BMS/Pfizer, Medtronic, Boehringer Ingelheim, Microlife, Roche and Daiichi-Sankyo. The other co-authors declared no conflict of interest.

## Supplementary Material

Supplementary Data
