# Corrigendum to: Risk of stroke in chronic heart failure patients with preserved ejection fraction, but without atrial fibrillation: analysis of the CHARM-Preserved and I-Preserve trials

**DOI:** 10.1093/eurheartj/ehx277

**Published:** 2017-06-01

*Eur Heart J* (2017) 38 (10): 742–750.

This paper has been retrospectively converted to Open Access, and the following funding statement has been added: ‘A-CP was supported by the Medical Research Council award MC_UU_12017/10.’

The paper has been corrected online.

