# Supplementary material for: Risk of stroke in chronic heart failure patients with preserved ejection fraction, but without atrial fibrillation: analysis of the CHARM-Preserved and I-Preserve trials
Source: Eur Heart J. 2016 Nov 13;38(10):742–50. doi: 10.1093/eurheartj/ehw509 (PMC5460584; doi:10.1093/eurheartj/ehw509)
Supplement: Supplementary Data [file ehw509_supp.docx]

**SUPPLEMENTAL MATERIAL**

Online Supplement for manuscript entitled:

**Risk of Stroke in Chronic Heart Failure Patients with Preserved Ejection Fraction and without Atrial Fibrillation: Analysis of the CHARM-Preserved and I-Preserve Trials.**

**Authors:** Azmil H. Abdul-Rahim, Ana-Cristina Perez, Rachael L. MacIsaac, Pardeep S. Jhund, Brian L. Claggett, Peter E Carson, Michel Komajda, Robert McKelvie, Michael R Zile, Karl Swedberg, Salim Yusuf, Marc A Pfeffer, Scott D. Solomon, Gregory Y.H Lip, Kennedy R. Lees, John J.V. McMurray.

*On behalf of the Candesartan in Heart failure Assessment of Reduction in Mortality and Morbidity- Preserved (CHARM-Preserved) and the Irbesartan in Heart Failure with Preserved Systolic Function (I-Preserve) steering committees.*

**Supplemental Tables:** I-V

**Supplemental Figures and Figure Legends:** I-VII

**Appendices:** I. Examples of risk score estimation using the model presented in Table 2.

II. List of the Candesartan in Heart failure Assessment of Reduction in Mortality and Morbidity- Preserved (CHARM-Preserved) and the Irbesartan in Heart Failure with Preserved Systolic Function (I-Preserve) steering committees members.

**SUPPLEMENTAL TABLES**

**Supplementary Table I.** Baseline characteristics according to atrial fibrillation (AF) status at baseline.

|  | **All patients**  **(N= 6701)** | | **Without AF**  (n= 4676) | **AF**  (n= 2025) |
| --- | --- | --- | --- | --- |
| ***Demographics,* n (%)** |  | |  |  |
| Age, year | 70 ±9 | | 69 ±9 | 72 ±8 |
| <65 | 1728 (26) | | 1400 (30) | 328 (16) |
| 65 - <75 | 2858 (43) | | 2032 (44) | 826 (41) |
| ≥75 | 2115 (32) | | 1244 (27) | 871 (43) |
| Race |  | |  |  |
| Caucasians | | 6212 (93) | 4273 (91) | 1939 (96) |
| Afro-American/Afro-Caribbean | | 190 (3) | 155 (3) | 35 (2) |
| Other | | 299 (5) | 248 (5) | 51 (3) |
| Female sex | 3576 (53) | | 2542 (54) | 1034 (51) |
| NYHA class |  | | | |
| II | 2461 (37) | | 1657 (35) | 804 (40) |
| III | 4085 (61) | | 2918 (62) | 1167 (58) |
| IV | 155 (2) | | 101 (2) | 54 (3) |
| Duration of heart failure, year |  | |  |  |
| < 2 year | 3989 (60) | | 2778 (59) | 1211 (60) |
| 2-5 year | 1557 (23) | | 1110 (24) | 447 (22) |
| > 5 year | 1116 (17) | | 764 (16) | 353 (17) |
| LV Ejection Fraction, % | 58 ± 9 | | 58 ± 9 | 58 ± 9 |
| ***Baseline vital signs*** |  | |  |  |
| BMI, kg/m^2^ | 30 ±6 | | 30 ±6 | 29 ±6 |
| BP, mmHg |  | |  |  |
| Systolic | 136 ±17 | | 137 ±16 | 135±17 |
| Diastolic | 78 ±10 | | 79 ±10 | 78 ±10 |
| Pulse pressure | 58 ±14 | | 58 ±14 | 58 ±14 |
| Heart rate, beats/min | 71 ±11 | | 71 ±11 | 73 ±12 |
| ***Laboratory measurements*** |  | |  |  |
| Serum creatinine, µmol/L | 90 ±30 | | 88 ±29 | 96 ±31 |
| Haemoglobin, g/ dL | 14 ±2 | | 14 ±2 | 14 ±2 |
| NT-proBNP*, pg/mL [(median (IQR)] | 339(134-964) | | 230(104-537) | 951(428-1698) |
| ***Medical history,* n (%)** |  | |  |  |
| Coronary heart disease | 3898 (58) | | 2960 (63) | 938 (46) |
| Myocardial infarction | 2025 (58) | | 1599 (34) | 426 (21) |
| Angina pectoris | 3298 (49) | | 2517 (54) | 781 (39) |
| CABG or PCI | 1377 (21) | | 1078 (23) | 299 (15) |
| Hypertension | 5342 (80) | | 3779 (81) | 1563 (77) |
| Diabetes mellitus | 1865 (28) | | 1313 (28) | 552 (27) |
| Stroke | 621 (9) | | 379 (8) | 242 (12) |
| ICD | 29 (0.4) | | 11 (0.2) | 18 (1) |
| Current smoker | 3707 (55) | | 2597 (56) | 1110 (55) |
| ***Medication,* n (%)** |  | | | |
| Diuretic (loop or thiazide) | 5160 (77) | | 3392 (73) | 1768 (87) |
| Loop diuretic | 3739 (56) | | 2278 (49) | 1461 (72) |
| Thiazide diuretic | 1912 (29) | | 1481 (32) | 431 (21) |
| ACE inhibitor | 1495 (22) | | 1020 (22) | 475 (24) |
| Aldosterone antagonist | 1285 (19) | | 788 (17) | 497 (25) |
| Beta-blocker | 3845 (57) | | 2761 (59) | 1084 (54) |
| Digitalis glycoside | 1250 (19) | | 405 (9) | 845 (42) |
| Calcium channel blocker | 2474 (37) | | 1809 (39) | 665 (33) |
| Anti-arrhythmic drug | 615 (9) | | 179 (4) | 436 (22) |
| Long-acting nitrate | 1948 (29) | | 1476 (32) | 472 (23) |
| Lipid lowering therapy | 2342 (35) | | 1786 (38) | 556 (28) |
| Antiplatelet therapy | 3985 (60) | | 3204 (69) | 781 (39) |
| Anticoagulant therapy | 1405 (21) | | 263 (6) | 1162 (57) |
| Any antithrombotic (antiplatelet or anti-coagulant therapy) | 5209 (78) | | 3408 (73) | 1801 (89) |
| Antidiabetic therapy (any) | 1531 (23) | | 1096 (23) | 435 (22) |
| Insulin therapy | 600 (9) | | 438 (9) | 162 (8) |
|  |  | |  |  |
| Placebo arm in the original trial | 3343 (50) | | 2322 (50) | 1021 (50) |

All continuous values are given in mean ± standard deviation unless stated otherwise. AF: atrial fibrillation; n(%): number of observations (percentage of observations within the group); BMI: body mass index; CABG: coronary artery bypass graft; PCI: percutaneous coronary intervention; ICD: implantable cardioverter defibrillator; ACE: angiotensin converting enzyme.

*Available in 3479 patients.

**Supplementary Table II.** Exploratory unadjusted univariable analysis for outcome of stroke in patients without AF.

| **Variables** | **HR (95% CI)** | **p-value** |
| --- | --- | --- |
| Age (per 10 year increase) | **1.48 (1.22-1.79)** | **<0.001** |
| Female sex | **0.72 (0.53-0.97)** | **0.029** |
| Heart rate (per 1bpm up to 70)* | 1.00 (0.98-1.03) | 0.881 |
| Systolic blood pressure (per 1mmHg increase) | **1.01 (1.00-1.02)** | **0.030** |
| LVEF (per 5% increase) | **0.91 (0.84-0.99)** | **0.033** |
| Creatinine (per 10 umol/L increase up to 350)^†^ | **1.09 (1.05-1.15)** | **<0.001** |
| BMI (per 5kg/m^2^ increase) | 0.89 (0.76-1.03) | 0.100 |
| NYHA class (III & IV vs. I & II) | 1.38 (0.98-1.94) | 0.067 |
| HF duration (≥2 years vs. <2 years) | 0.91 (0.67-1.25) | 0.565 |
| Current smoker | 0.80 (0.59-1.08) | 0.147 |
| Coronary heart disease (angina, MI, revascularisation, CABG, IHD) | 1.01 (0.74-1.37) | 0.962 |
| Previous Stroke | **3.23 (2.24-4.67)** | **<0.001** |
| Hypertension | 1.30 (0.85-2.01) | 0.229 |
| Insulin treated diabetes | **2.25 (1.51-3.36)** | **<0.001** |
| NT-proBNP, pg/mL (log)^‡^ | **1.48 (1.27-1.73)** | **<0.001** |

Significant level at conventional p<0.05 in bold. LVEF indicates left ventricular ejection fraction; BMI: body mass index; NYHA: New York Heart Association; MI: myocardial infarction; CABG: coronary artery bypas graft; IHD: ischaemic heart disease.

* Heart rate was truncated to 70bpm to avoid co-linearity with possible atrial fibrillation.

^†^ The values were truncated to the level displayed due to individual variable’s non-linearity.

^‡^ Univariable analysis for log NT-ProBNP was performed for patients with NT-ProBNP measurements only.

**Supplementary Table III.** “HF-PEF model for stroke” derived from HF-PEF cohort, without AF.

| **Variables** | **Hazard ratio** | **Lower 95%CI** | **Upper 95%CI** | **Χ^2^-value (Χ^2^=82.8)** | **Coefficients** |
| --- | --- | --- | --- | --- | --- |
| Previous Stroke | 2.92 | 2.02 | 4.23 | 32.4 | 1.071 |
| Age (per 10 year increase) | 1.63 | 1.34 | 1.97 | 21.6 | 0.450 |
| Diabetes treated with insulin | 2.52 | 1.68 | 3.78 | 19.9 | 0.923 |
| Sex (Male) | 1.60 | 1.17 | 2.16 | 8.9 | 0.465 |

See the appendix for explanation of how to use coefficients to predict individual patient’s risk of stroke.

**Supplementary Table IV.** Validation of stroke model using TOPCAT for patients without AF (n=2205)

| **Variables** | **Hazard ratio** | **Lower 95%CI** | **Upper 95%CI** | **P-value** | **Coefficients derived from CORONA-GISSI** |
| --- | --- | --- | --- | --- | --- |
| Previous Stroke | 2.49 | 1.12 | 5.53 | 0.026 | 0.591 |
| Diabetes treated with insulin | 1.90 | 1.04 | 3.45 | 0.036 | 0.626 |
| BMI (per 5kg/m^2^ increase up to 30) | 0.84 | 0.49 | 1.45 | 0.529 | -0.301 |
| Age (per 10 year increase) | 1.09 | 0.81 | 1.46 | 0.582 | 0.331 |
| NYHA class (NYHA III and IV) | 1.02 | 0.55 | 1.86 | 0.959 | 0.472 |

See the appendix for explanation of how to use to predict individual patient’s risk of stroke. BMI: body mass index; NYHA: New York Heart Association.

**Supplementary Table V: *Sensitivity analysis***- Comparison of “stroke in HF-REF” model’s discrimination within HF-PEF cohort, using the Overall C-Index and the traditional Harrell’s C.

|  | **Overall C-Index***  C-index (95%CI) | **Harrell’s C^†^**  C-index (95%CI) |
| --- | --- | --- |
| ‘Stroke in HF-REF’ model applied to HF-PEF cohort^‡^ | 0.73 (0.59-0.85) | 0.65 (0.60-0.69) |
| ‘Stroke in HF-REF’ model validated in TOPCAT data | 0.86 (0.62-0.99) | 0.64 (0.50-0.66) |

* Overall C-Index is calculated according to Pencina *et al* method (Ref. 16), as outlined by Liu *et al* (Ref. 17).

^†^ Harrell’s C is calculated using Harrell *et al* method (Ref. 18).

^‡^ Variables for ‘Stroke in HF-REF model’ are outlined in Table 2 of the main text.

*Commentary:* Harrell’s method gave lower C-indices (as compared to the Overall C-Index) although the 95% CI for both methods overlapped.

**SUPPLEMENTAL FIGURES AND FIGURE LEGENDS**

**Supplementary Figure I.** Kaplan Meier curves for stroke in HF-PEF patients with atrial fibrillation, according to anticoagulant treatment. HF-PEF indicates heart failure with preserved ejection fraction; AF, atrial fibrillation.

**
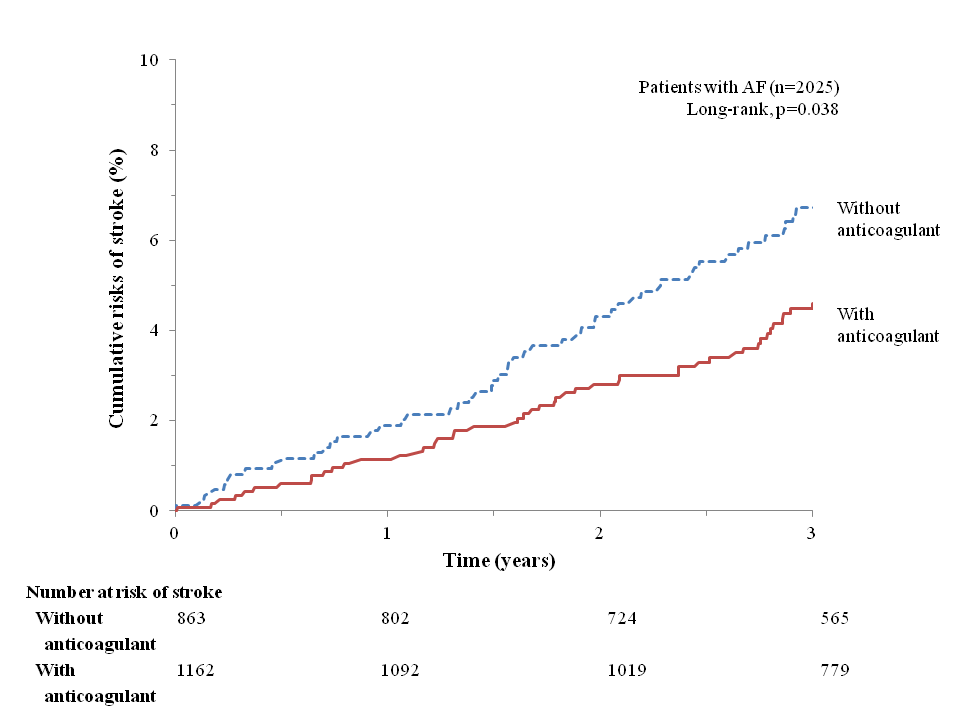
**

*Commentary:* In patients with AF treated with an anticoagulant, the 1, 2, and 3 year KM rates of stroke were 1.1 (95%CI: 0.7-2.0), 2.8 (95%CI: 2.0-4.0), and 4.5 (95%CI: 3.4-5.9) %, respectively; the corresponding KM rates for patients with AF not treated with an anticoagulant were 1.9 (95%CI: 1.2-3.1), 4.3 (95%CI: 3.1-6.0), and 6.7 (95%CI: 5.2-8.8) %, respectively.

**Supplementary Figure II.** Validation using TOPCAT, for patients without AF: Distribution of the risk score for stroke.


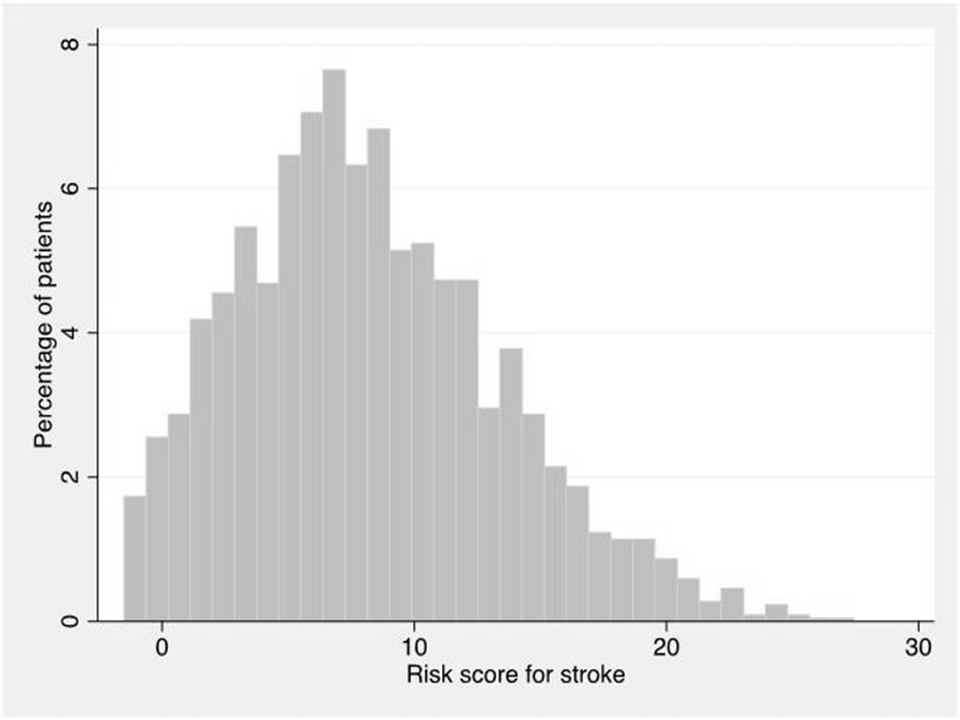


**Supplementary Figure III.** Kaplan-Meier plot for stroke by tertiles of their risk scores in patients without AF (using TOPCAT, for patients without AF).

**
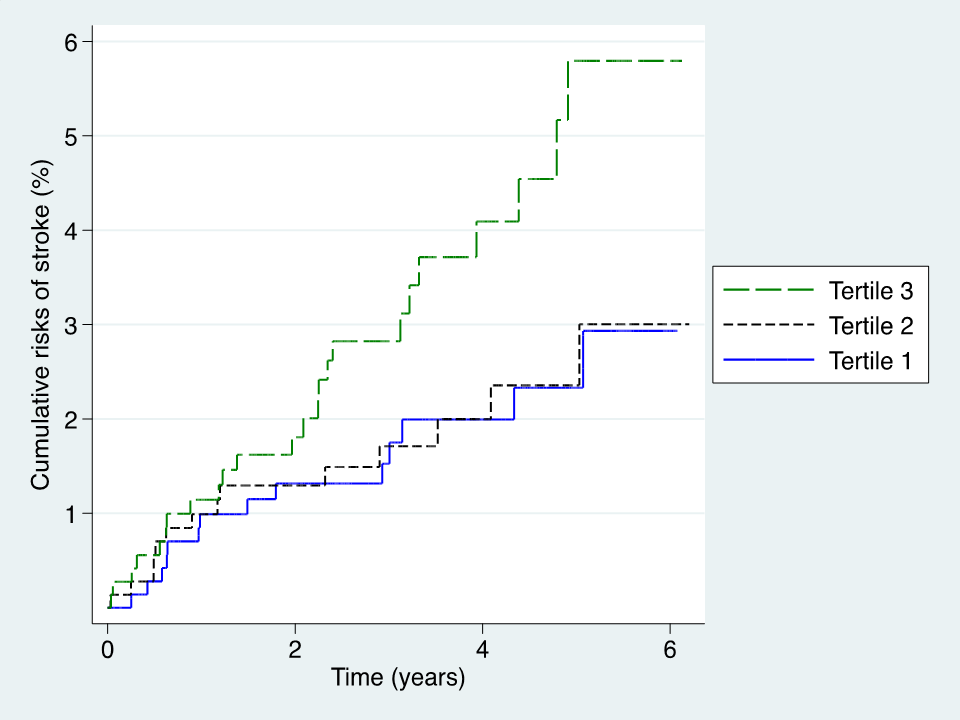
**

**Supplementary Figure IV.** Cumulative incidence function for stroke in HF-PEF patients with atrial fibrillation, according to anticoagulant treatment at baseline (considering death as a competing risk).

HF-PEF indicates heart failure with preserved ejection fraction; AF, atrial fibrillation.

**
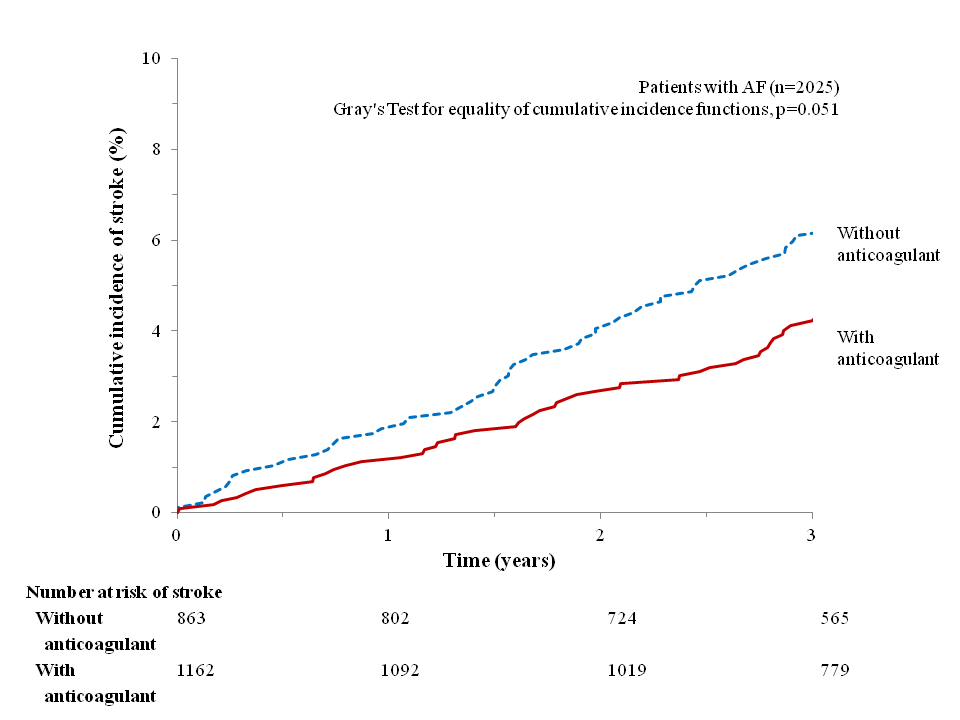
**

**Supplementary Figure V.** Cumulative incidence function for stroke in patients with HF-PEF according to AF status at baseline (with death as a competing risk).

AF indicates atrial fibrillation, HF-PEF: heart failure with preserved ejection fraction.

**
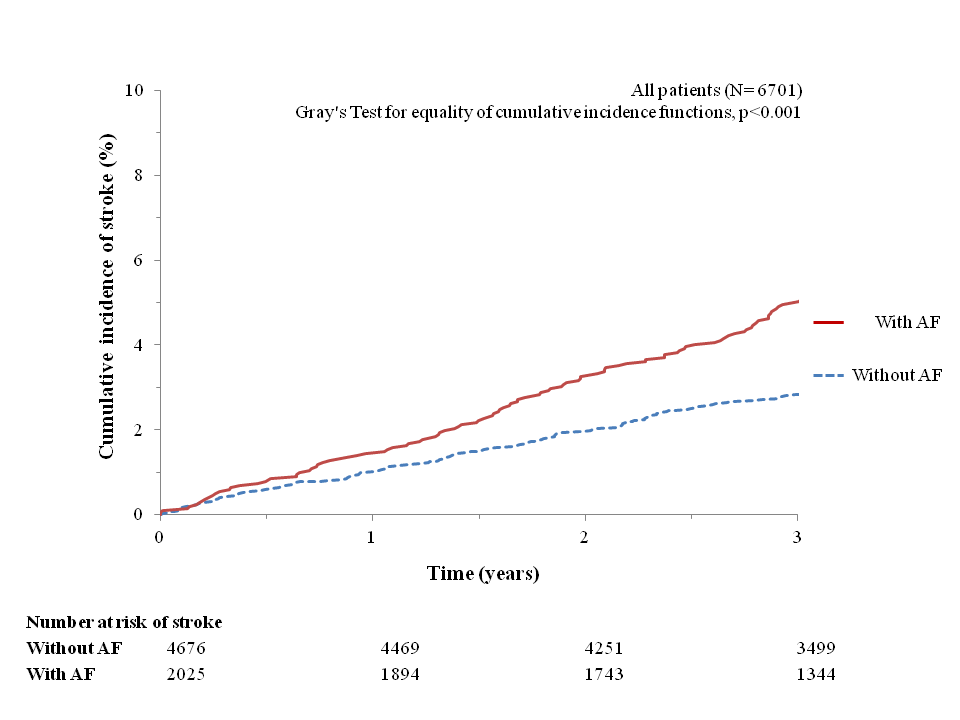
**

**Supplementary Figure VI.** Cumulative incidence function plot for stroke according to tertile of risk score in patients without AF (with death as a competing risk).

AF indicates atrial fibrillation.

**
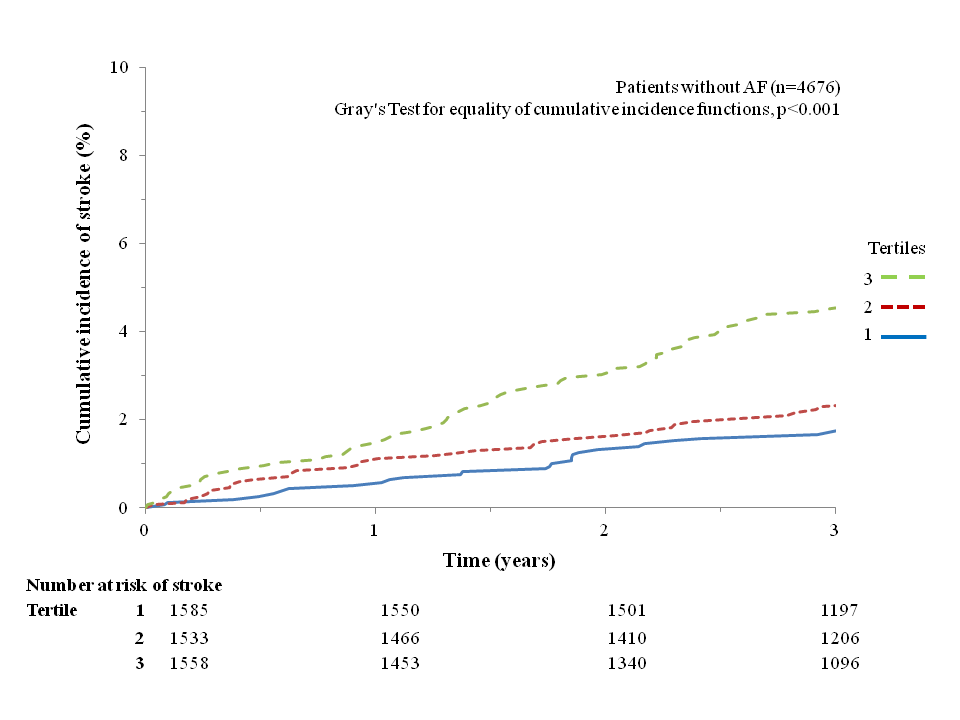
**

**Supplementary Figure VII.** Cumulative incidence function plot for stroke by tertiles of their risk scores in patients without AF (using TOPCAT, for patients without AF- accounting death as competing risk).


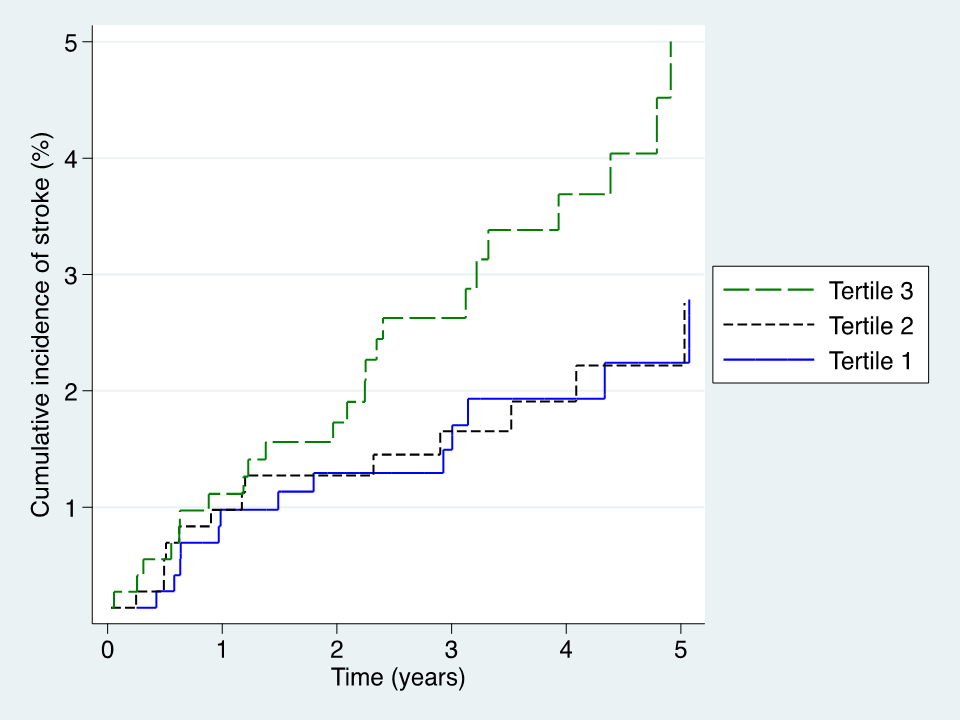


**APPENDICES**

**Appendix I. Examples of risk score calculation using the model presented in Table 2.**

This example illustrates the use Table 2 and associated Figures 3 - 4, to calculate the risk score of stroke in individual patients.

For example, consider a patient aged 70 years in NYHA functional class II with a BMI of 25 kg/m^2^ and had a previous stroke. Using the HF-REF model coefficients in Table 2, each multiplied by 10, this patient’s risk score for stroke is: (3.31 x 7) + [(-3.01) x 5] + 5.91 = 14.03. Note that age is in decades, hence 70 becomes 7; BMI is in steps of 5, BMI of 25 becomes 5.

**Appendix II. List of the Candesartan in Heart failure Assessment of Reduction in Mortality and Morbidity- Preserved (CHARM-Preserved) and the Irbesartan in Heart Failure with Preserved Systolic Function (I- Preserve) steering committees members.**

CHARM- Preserved group: Salim Yusuf, Marc A. Pfeffer, Karl Swedberg, Christopher B. Granger, Peter Held, John J. V. McMurray, Eric L. Michelson, Bertil Olofsson, and Jan Östergren.

I- Preserve group: Barry M. Massie, Peter E. Carson, John J. McMurray, Michel Komajda, Robert McKelvie, Michael R. Zile, Susan Anderson, Mark Donovan, Erik Iverson, Christoph Staiger, and Agata Ptaszynska.
